# A Neglected Method

**Published:** 1892-06

**Authors:** 


### A NEGLECTED METHOD.

At a recent meeting of the New York Surgical Society, Dr. Charles McBurney read a paper entitled "A Neglected Method of Modifying General Anæsthesia." The method consists in placing a broad, elastic tourniquet around each of the thighs as well as around the upper limbs before the etherization is commenced, thus shutting off from the general circulation a considerable portion of the blood. He has employed this method about ten times at the Roosevelt Hospital, with very good results. The patient is usually brought under the influence of the ether in a very short time—in most of the cases, within three or four minutes. The trembling of the limbs, so annoying to the operator, especially in operations about the perineum, is entirely absent. The patients usually recover from the effects of the anæsthetic within two or three minutes after the tourniquets are loosened, and in no case has vomiting occurred. Some precautions are necessary. In case a hypodermic is given before the ligatures have been removed, the quantity of the drug must be considerably smaller than the usual dose. The ligatures are first applied loosely so as to retard only the venous blood; then when the limbs are surcharged, the bandages are tightened so as to completely stop the arterial circulation as well. Dr. McBurney then brought a man before the society and gave a practical illustration of the method. He stated that until a short time ago he thought the method a new one; since then, however, he has found it advocated in a letter to the New York Medical Journal, published in 1887, by Dr. Leonard Corning, of New York, as well as by others. He had, therefore, changed the title of his paper and called it "A Neglected Method" instead of a New Method.—*Medical Age.*

